# Urgent need to modernize pharmacovigilance education in healthcare curricula: review of the literature

**DOI:** 10.1007/s00228-018-2500-y

**Published:** 2018-06-20

**Authors:** Michael Reumerman, J. Tichelaar, B. Piersma, M. C. Richir, M. A. van Agtmael

**Affiliations:** 1 Pharmacotherapy Section, Department of Internal Medicine, Amsterdam UMC, Amsterdam, The Netherlands; 2 Research and Expertise Centre in Pharmacotherapy Education (RECIPE), Amsterdam, The Netherlands

**Keywords:** Medical education, Pharmacotherapy, Pharmacovigilance

## Abstract

**Objectives:**

Pharmacovigilance education is essential since adverse drug reactions (ADRs) are a serious health problem and contribute to unnecessary patient burden and hospital admissions. Healthcare professionals have little awareness of pharmacovigilance and ADR reporting, and only few educational interventions had durable effects on this awareness. Our future healthcare providers should therefore acquire an adequate set of pharmacovigilance competencies to rationally prescribe, distribute, and monitor drugs. We investigated the pharmacovigilance and ADR-reporting competencies of healthcare students to identify educational interventions that are effective in promoting pharmacovigilance.

**Methods:**

The PubMed, EMBASE, Cochrane, CINAHL, PsycINFO, and ERIC databases were searched using the terms “pharmacovigilance,” “students,” and “education.”.

**Results:**

Twenty-five cross-sectional and 14 intervention studies describing mostly medical and pharmacy students were included. Intentions and attitudes on ADR reporting were overall positive, although most students felt inadequately prepared, missed the training on this topic, and lacked basic knowledge. Although nearly all students observed ADRs during clinical rounds, only a few had actually been involved in reporting an ADR. Educational interventions were predominately lectures, sometimes accompanied by small interactive working groups. Most interventions resulted in a direct increase in knowledge with an unknown long-term effect. Real-life learning initiatives have shown that healthcare students are capable of contributing to patient care while increasing their ADR-reporting skills and knowledge.

**Conclusions:**

There is an urgent need to improve and innovate current pharmacovigilance education for undergraduate healthcare students. By offering real-life pharmacovigilance training, students will increase their knowledge and awareness but can also assist current healthcare professionals to meet their pharmacovigilance obligations.

**Electronic supplementary material:**

The online version of this article (10.1007/s00228-018-2500-y) contains supplementary material, which is available to authorized users.

## Introduction

Most healthcare students enter clinical practice immediately after graduation and are required to prescribe, distribute, administer, and/or monitor drugs on a daily basis. In order to perform these responsibilities effectively and to ensure the safe use of medications, healthcare students (especially in medicine, pharmacy, dentistry, and nursing curricula) should acquire a minimum set of pharmacovigilance competencies before they graduate and start clinical practice [1, 2]. Foreseeing, recognizing, managing, and reporting adverse drug reactions (ADRs) are an important part of rational and safe prescribing and are integrated into multiple steps of the WHO-six-step Guide to Good Prescribing [3]. It is a professional responsibility of all healthcare professionals. Despite this, healthcare curricula often teach little on pharmacovigilance and ADR reporting, with a median of 4–5.5 contact hours [4]. Numerous studies have expressed concern about the lack of healthcare professional competencies in pharmacovigilance [4–6].

This lack of undergraduate education and training in pharmacovigilance is consistent with the low level of knowledge, skills, and actions seen not only in physicians but also in practicing pharmacists, dentists, and nurses [7–9]. Unfamiliarity with pharmacovigilance, a low level of ADR-reporting skills, a lack of knowledge combined with negative attitudes like ignorance, fear legal liability, and lack of importance are thought to be related to the current inadequate response to many ADRs [10–13]. Several interventions (implementing protocols, educational workshops, or repeated emailing or telephone calls) have been implemented in an attempt to improve the competence of healthcare professionals [14–17], but these interventions are costly or fail to produce clinically relevant and long-term effects [8].

Despite the urgency of this problem, each year millions of medication users experience ADRs ranging from minor discomfort to hospital admission, permanent disability, or even death [18]. ADRs are responsible for 3.0–6.5% of all hospital admissions, 0.15% of all deaths, and could have been prevented in 47–72% of cases by good pharmacological and pharmacovigilance skills and knowledge [19–22].

Pharmacovigilance centers have an important role in the dissemination of current pharmacovigilance knowledge. Their data are mainly based on post-marketing reporting, which is essential for identifying previously undetected, uncommon, or serious ADRs. In most countries, pharmacovigilance center causality assessments of ADRs rely on a mixture of spontaneous reporting by healthcare professionals (physicians, pharmacists, nurses, and dentists) and patients. Since healthcare professionals have a different focus in ADR reporting, it is important to involve all parties [23–27]. With population aging, the increased use of prescription drugs and polypharmacy will probably lead to a drastic rise in the number of ADRs [28]. This together with ADR underreporting [29, 30] and the lack of awareness and understanding of ADRs could lead to an even greater burden on patients and healthcare systems in the near future.

By studying the pharmacovigilance and ADR-reporting competencies of healthcare students, we aim to identify effective educational interventions that promote pharmacovigilance early in their education and career. The primary objectives of this review were therefore to analyze the following: (1) what is known about the pharmacovigilance competencies of healthcare students and (2) which educational interventions are effective in pharmacovigilance education.

## Methods

### General methodology

We searched the literature to analyze the current level of competencies and the effects of different undergraduate pharmacovigilance interventions, using the Kirkpatrick model of hierarchy of evaluation, as modified by Freeth [31]. Given the diverse outcome measures, no meta-analysis was performed.

### Search strategy

With assistance of a medical information specialist (R.O.), the MEDLINE (PubMed), EMBASE, PsycINFO, Cochrane, CINAHL, and ERIC databases were searched for articles on pharmacovigilance education. MEDLINE was used as the standard medical research database. The Embase, PsycINFO, Cochrane, and CINAHL databases were used for articles published in biomedical and nursing databases. The ERIC database functioned as a supplementary detector for educational articles. All databases were searched until February 1, 2017, with database-specific queries [S4] without additional filters. All queries used “pharmacovigilance,” “students,” and “education” or commonly used abbreviations of similar terms (e.g., adverse drug reporting systems, undergraduate, and teaching, respectively). Articles were retrieved from the local university library or requested from the original authors, institution, or publisher. The references of relevant articles were screened using the snowball method [32].

### Study selection

First, two authors (MR and BP) independently screened all articles for eligibility based on their titles and preset inclusion and exclusion criteria [Supplement Table 1]. If there was any discrepancy about the content of the article, the abstract (if available) and/or full article was screened. Disagreements were resolved by mutual consensus. All eligible abstracts and articles were assessed in a similar way. Articles were included if they analyzed pharmacovigilance competencies in undergraduate healthcare students. Articles were not limited to the study setting, country of origin, or publication date. Exclusion criteria were as follows: (1) outcome measure not related to the pharmacovigilance competencies; (2) evaluation of a specialty-specific ADR; (3) undergraduate healthcare students were not studied (e.g., healthcare professionals or patients); (4) language other than English or Dutch; (5) studying medical or dietary supplements, herbal products, or alternative medicines; and (6) non-original research studies (e.g., reviews, editorials, letters to the editor, and conference abstracts).

### Data extraction

Data were extracted by two authors [MR and BP] using a modified coding sheet, based on the Best Evidence Medical Education (BEME) Collaboration coding sheet [33, 34]. This modified coding sheet included the study design and aim, instruments used, characteristics of the educational intervention, students’ educational level and performance, overall conclusion, and recommendations. The Kirkpatrick model of hierarchy of evaluation, modified by Freeth [31], was added to evaluate the outcome level.

### Quality assessment

Study quality was assessed using the Medical Education Research Study Quality Instrument (MERSQI) [35]. This instrument was developed to assess educational studies and consists of six domains: study design, sampling, type of data, validity of the evaluation instrument, data analysis, and outcomes. Scores range from 5 to 18 points. Although there is no defined cutoff for high- or low-quality study methods, a previous study considered scores of 5–8.5 to reflect a low-quality study method, 9.0–13.0 to reflect a moderate-quality study method, and 13.5–18 as a high-quality study method [36].

### Data analysis

Data were analyzed using SPSS Statistics 22 (Chicago, IL). Descriptive statistics were used to report total mean MERSQI scores, proportion of articles with a different country of origin, type of healthcare student, and study design. The MERSQI scores of the main groups of student outcomes were compared using a one-way ANOVA with an alpha of < 0.05.

Given the differences in study design and outcome measures, only a quantitative analysis was possible. Student motives for reporting ADRs were described using descriptive statistics. Student opinions on educational aspects were recoded into three groups (No: ≤ 33% of students (fully) agreed, Neutral: ≥ 34 ≤ 66% of students (fully) agreed, Yes: ≥ 67% of students (fully) agreed.

## Results

### Search results

**Fig. 1:**
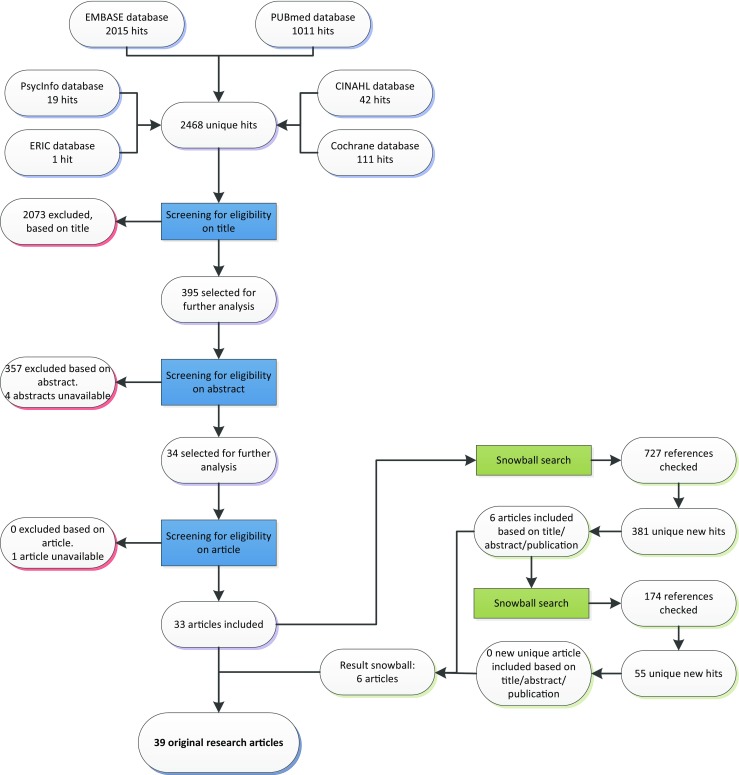
Flow diagram of article selection. In green, the snowball search is highlighted

The initial search identified 2468 unique articles. Figure 1 shows the flowchart of the search, selection, and review process. Thirty-three articles were eligible for inclusion. The 727 references of the 33 eligible articles were snowball searched, which yielded 6 new articles. In total, 14 intervention and 25 cross-sectional articles were included in our analysis.

### Acquired pharmacovigilance competencies

As shown in Table 1, there is no uniform pharmacovigilance evaluation method. Most articles studied ADR reporting and pharmacovigilance knowledge (Kirkpatrick level 2b) in undergraduate medical and pharmacy students. Two studies [41, 42] used identical research and outcome measures and have been compared separately [61].

**Table 1:** Overview of published (*n* = 25) pharmacovigilance cross-sectional studies in undergraduate healthcare students

Author	Setting	Student type	Total students	Methods	Main results	Kirkpatrick level^a^	MERSQI score
Ahmad et al. [37]	India, 4 private pharmacy schools	Pharmacy (fourth to fifth year PharmD/BPharm)	284	21-point self-administered questionnaire on attitudes and knowledge	PharmD considered themselves better trained (73.8 vs 23.8%), and more students express concerns about authorities not working together (93.4 vs 74.0%). Significant higher knowledge score in PharmD (6.98 ± 1.79; 0–11 min/max) compared to BPharm (4.25 ± 1.82).	2b	14.5
Rajiah et al. [38]	Malaysia, 1 private medical school	Pharmacy (fourth year BPharm)	108	24-point survey questionnaire on knowledge and perceptions	Male students felt significantly more prepared to report ADRs (*p* = 0.040) in their future practice. Males knew more about post-marketing surveillance (*p* = 0.003) where females knew more about the causality assessment of ADRs (*p* = 0.045).	2b	12.5
Saurabh et al. [39]	India, 1 college-associated hospital	Medical (sixth year)	68	20-point questionnaire on knowledge, attitudes, and practice (KAP)	All students knew the term “pharmacovigilance” and were aware where to report ADRs. The majority (85.29%) had never reported before.	2b	9.5
Schutte et al. [40]	Netherlands, 8 medical schools	Medical (third to sixth year)	874	10-question (with multiple statements) e-questionnaire on intentions, attitudes, skills, and knowledge	Students intended (18.27 ± 2.74; 3–21 min/max) and planned (4.95 ± 1.23; 1–7 min/max) to report ADRs and had a higher intention score (*p* = 0.04) if they had reported an ADR before. Between 65.4 and 82.1% knew why ADRs should be reported, 35.5–77.6% did not know where to report, and 65.9–89.7 did not know which items were necessary for a good ADR report. Sixth-year students were significantly (*p* < 0.05) more knowledgeable than third-year students.	2b	12.5
Abubakar et al. [41]	Nigeria, 1 university	Medical (fourth to fifth year)	108	25-point survey questionnaire on knowledge, attitudes, and practice (KAP)	95% felt ADR monitoring benefits patients while 84% felt ADR reporting is time-consuming with no outcome. 93% believed all marketed drugs are safe and 90% were not aware of any nearby pharmacovigilance centers. 99% have come across an ADR; however, only 4% has ever reported an ADR.	2b	12
Abubakar et al. [42]	Malaysia, 1 university	Medical (fourth to fifth year)	87	25-point survey questionnaire on knowledge, attitudes, and practice (KAP)	87% agreed ADR reporting is a professional obligation, and most (74%) disagreed ADR reporting is time-consuming with no outcome. About half of students knew the definitions of ADR (68%) or pharmacovigilance (49%) or the functions of pharmacovigilance (59%). 85% were not aware of any nearby pharmacovigilance center. 72% had come across an ADR and only 1% had ever reported an ADR.	2b	12
Farha et al. [43]	Jordan, 3 universities	Pharmacy (fourth to sixth year PharmD/BPharm)	434	26-point survey questionnaire on knowledge and perceptions	65% were willing to report ADRs during their clerkships. 73.2% agreed pharmacovigilance should be made compulsory for pharmacists. Overall poor knowledge score (4.0; 0–10 min/max). PharmD (5.4 ± 2.3) students or attending a previous pharmacovigilance course (6.1 ± 1.9) showed significant (*p* < 0.001) higher knowledge score. Sixth-year students had a significant lower score than fifth-year students (3.0 ± 1.7 vs 4.3 ± 2.3).	2b	13
Ismail et al. [44]	Malaysia, 1 university	Medical (fifth year)	23	12-point questionnaire on perceived knowledge	All students think ADRs should be reported. 87% have witnessed an ADR before; however, only 8.7% perceived to know about the pharmacovigilance program. 87% think good knowledge of undergraduate pharmacology would have improved the ADR reporting skills.	2a	9
Meher et al. [45]	India, tertiary care teaching hospital	Medical (second, fourth, and fifth year)	180	21-point questionnaire on knowledge, attitudes, and practice (KAP)	Overall knowledge scores [pre-final (5.63 ± 1.79; 0–10 min/max) vs final (4.76 ± 1.57)] and attitude scores [pre-final (4.95 ± 1.34; 0–7 min/max) vs final (4.26 ± 0.79)] were significantly higher in pre-final year students. Practice scores (1.66 ± 0.79; 0–4 min/max) were highest for final year students, however non-significant.	2b	10.5
Shalini et al. [46]	Malaysia, private university	Dentistry (fourth to fifth year)	62	29-point survey questionnaire on attitude and knowledge	Most students (96.9–99.6%) agreed ADR reporting is necessary. 24.6% knew the definition of pharmacovigilance and 34.4% knew the purpose of pharmacovigilance. No student knew the regulatory body and only 3.3% knew which ADR reporting system is currently used. Final year students had higher knowledge scores (20.44 vs 11.03; unknown max); however, pre-final year students had better attitude scores (32.35 vs 25.40; unknown max).	2b	13
Umair Khan et al. [47]	Pakistan, 1 University	Medical + pharmacy [PharmD] (fifth to sixth year)	199	29-point self-administered questionnaire on knowledge, attitudes, and perceptions	More pharmacy students found ADR reporting as important as managing patients (79.1 vs 43.5%); however, both believed it was their responsibility (98.9 vs 92.5%) to report. Pharmacy (5.61 ± 1.78; 0–10 min/max) showed significantly higher knowledge scores compared to medical students (3.23 ± 1.60). Previous experience with or exposure to ADRs showed a non-significant (*p* = 0.156) higher knowledge score (4.54 ± 2.04 vs 4.02 ± 1.85).	2b	13.5
Iffat et al. [48]	India, different (n = ?) private and public universities	Medical + dentistry (third to fifth year)	531	31-point questionnaire on perceived knowledge and attitudes	53.29% felt ADR reporting was a professional obligation; however, only 26.55% had witnessed an ADR, 9.79% perceived to know where to report, and 8.85% perceived to know how to report and ADR. Final-year students were significantly more familiar with most knowledge questions. No analysis between curricula was done.	2a	11
Showande et al. [49]	Nigeria, 1 university	Pharmacy (fourth to fifth year)	69	Questionnaire on knowledge, personal experiences, and opinions on current ADR-reporting guidelines	21.7% had claimed to have seen the ADR reporting form; however, only 6.7% could actually name the correct color of this form. Students (strongly) agreed that pharmacists, physicians, and nurses were the 3 most important healthcare professionals who should report ADRs.	2b	12.5
Gavaza et al. [6]	USA, 1 college of pharmacy	Pharmacy (third year PharmD)	58	58-point survey questionnaire on intention, attitude, and knowledge of ADE reporting	Student intended to report (5.9 ± 1.9; 1–7 min/max), would try to report (6.0 ± 1.3), and planned (5.8 ± 1.3) to report serious ADRs. Knowledge on what/when to report: all ADRs (37.9%), missing details (51.7%) uncertainty about the cause (58.6%) was difficult. A streamlined MedWatch form, clear knowledge of what constitutes a reportable ADE, and employer support of ADE reporting would make reporting easier.	2b	12.5
Hema et al. [50]	India, 1 medical college	Medical (fifth to sixth year)	210	25-point questionnaire on awareness, knowledge, and method of application	Overall awareness, knowledge, and method of application were significantly lower among students (A 2.45 ± 1.24; 0–5 min/max, K 2.3 ± 1.27, and M 3.18 ± 2.19) than among interns (A 3.06 ± 1.07, K 3.20 ± 1.62, and M 5.65 ± 2.22) and postgraduates. Knowledge was positively correlated with the method of application in the total group (*r* 0.485, *p* < 0.001)	2b	12
Sharma et al. [51]	India, 5 colleges from technical and public universities	Pharmacy (BPharm fourth year)	180	Questionnaire on knowledge and awareness	37.8% had no idea of how to report an ADR and 90% did not know about the regulatory body. 76.6% believed pharmacists to be the most important healthcare professional to report; however, only 37.1% had reported an ADR before.	2b	9.5
Vora et al. [52]	India, 6 medical colleges	Medical (second to third year)	880	18-point questionnaire on pharmacovigilance and ADR-related knowledge	Overall knowledge scores for ADRs: (1.26 ± 1.24: 0–9 min/max–3.18 ± 1.72 were significantly higher than scores for pharmacovigilance: 0.40 ± 0.69: 0–9 min/max–2.43 ± 1.86). In most universities, third-year students had a significantly lower ADR and pharmacovigilance knowledge score than second-year students.	2b	13.5
Elkalmi et al. [53]	Malaysia, 5 universities	Pharmacy (fourth year)	510	25-point survey questionnaire on knowledge and perceptions	75.6% felt ADR reporting should be made compulsory and 90.4% felt pharmacists are one of the most important healthcare professionals to report ADRs. Overall high knowledge scores 6.9 ± 1.4 (0–10 min/max) which were significantly higher (*p* < 0.01) after a pharmacovigilance course (7.1 ± 1.2 vs 6.7 ± 1.5).	2b	14.5
Sears et al. [54]	USA, 9 colleges of pharmacy	Pharmacy (third to sixth year)	1322	26-point digital survey questionnaire on knowledge, skills, practice, and learner methods	Students from all academic years were more aware of reporting to MedWatch (13.4–91.6%) than VAERS (10.5–68.2%) and MER (20.2–57.4%). Sixth-year students were significantly (*p* < 0.001) more knowledgeable about all ADR systems; however, only 56.1% of sixth-year students were able to locate forms, 12.1% were able to complete a form, and 39.3% demonstrated understanding of the MedWatch program. Most students cited “The didactic curriculum” and “experimental rotations” as mechanisms of learning.	2b	11.5
Rehan et al. [55]	India, 1 medical college	Medical (fifth year)	107	11-point questionnaire on knowledge, attitudes, and practices	98% agreed ADR monitoring should be done routinely; however, only 61.6% knew the spontaneous reporting, and 58.9% knew the intensive monitoring method. Of all students, only 7 (6.5%) could correctly define an ADR.	2b	9.5
Sivadasan et al. [56]	Malaysia, 1 private university	Nursing (third to fourth year)	32	29-point survey questionnaire on knowledge and attitudes	All pre-final students (strongly) agreed that ADR reporting is necessary and a professional obligation; however, only 76.2% of final-year students (strongly) agreed. 18.8% knew the purpose of pharmacovigilance, and 37.5% knew the definition of ADR; however, only 9.4% knew the regulatory body for ADR reporting.	2b	12.5
Sivadasan et al. [57]	Malaysia, 1 private university	Medical + pharmacy (third to fifth year)	271	28-point survey questionnaire on knowledge and perceptions	Final-year pharmacy students had a significant higher knowledge score than medical students (8.4 ± 0.2; 0–15 min/max vs 3.17 ± 0.06); however, pre-final year medical students were more knowledgeable than pre-final year pharmacy students (5.12 ± 0.06 vs 3.84 ± 0.02). More final and pre-final year medical students respectively strongly agreed ADR reporting is necessary (73.1/80.5% vs 69.0/75.8%) and their professional obligation (50.0/69.5% vs 54.8/51.6%).	2b	13
Isfahani et al. [58]	Iran, 1 university	Pharmacy (third to fifth year)	71	17-point questionnaire on knowledge attitude and practice (KAP)	88.68% (completely) agreed that ADR reporting is a duty of all healthcare professionals and 83.09% think educational programs have positive effects on ADR reporting. 30.98% were aware of the national pharmacovigilance center and program; however, only 4.28% have reported any ADRs.	2b	7.5
Limuaco et al. [59]	Philippines, 1 university	Pharmacy (fourth year)	?	Questionnaire on perceived awareness, knowledge, and attitudes	Students had high level of awareness about pharmacovigilance, ADRs, and adverse drug events (mean 4.01 ± 0.25; 1–5 min/max) and were reasonably familiar with ADR monitoring, reporting, and documentation (mean 3.53 ± 0.24; 1–5 min/max); however, most had neutral attitudes about education and training during their curriculum (mean 3.31 ± 1.32; 1–5 min/max).	2a	6
Rosebraugh et al. [60]	USA, 79 internal medicine clerkships	Medical (third year)	?	Questionnaire on opinions and attitudes of available courses on Clinical Pharmacology	47% of schools had clinical rotations that included clinical pharmacology or ADR training; however, only 8% was mandatory. The elective courses mainly offered 11 h of didactic lectures. 61% believed an educational training of high quality would be of value.	2a	7

*PharmD*, Doctor of Pharmacy; *BPharm*, Bachelor of Pharmacy; *ADRs*, adverse drug reactions; *ADE*; adverse drug event; *VAERS*, Vaccine Adverse Event Reporting System

^a^Kirkpatrick’s four levels of training evaluations are as follows: Level 1—participation, covers learners’ views on the learning experience, its organization, presentation, content, teaching methods, and aspects of the instructional organization, materials, and quality of instruction; Level 2a—modification of attitudes and perceptions: outcomes relate to changes in the reciprocal attitudes or perceptions between participant groups toward the intervention or simulation; Level 2b—modification of knowledge or skills: for knowledge, this relates to the acquisition of concepts, procedures, and principles; for skills, this relates to the acquisition of thinking problem solving, psychomotor, and social skills; Level 3—behavioral change: documents the transfer of learning to the workplace or willingness of learners to apply new knowledge and skills; Level 4a—change in organizational practice: wider changes in the organization or delivery of care, attributable to an educational program; Level 4b—benefits to patient or clients: this relates to any improvement in the health or well-being of patient clients as a direct result of an educational program

Twenty-two articles analyzed student opinions, intentions, and attitudes to ADR reporting and pharmacovigilance. Between 53 and 100% of students agreed that ADR reporting was a professional responsibility [42, 46–48], and most articles concluded that pharmacists were the most important healthcare professionals for this [37, 43, 47, 51]. However, all students agreed that all healthcare professionals should be aware of ADRs and ADR reporting [49, 58]. Students had favorable intentions about reporting ADRs (5.9 ± 1.5 to 6.17 ± 0.95; 1–7 min/max) and would try to report (6.0 ± 1.3 to 6.10 ± 1.0; 1–7 min/max) serious ADRs during their internships/clerkships [6, 40]. A large proportion (73.5–75.6%) of students agreed that ADR reporting should be compulsory for pharmacists [39, 43, 53].

Almost all articles analyzed students’ knowledge to some degree, although skills were not analyzed in the cross-sectional studies. Overall knowledge was poor, since only half (37.5–80%) of the students were familiar with the term “adverse drug reactions” [37, 41, 42, 45–47, 53, 57], “pharmacovigilance” (18–66%) [41, 42, 45–47, 53, 57], and the clinical relevance of pharmacovigilance (19–63%) [41, 42, 46, 57]. In contrast, students’ knowledge of the ADR classification of Rawlins [62], a more challenging topic, was known in these two studies [47, 53].

Fourteen articles analyzed what students did in practice in terms of pharmacovigilance and ADR reporting. Although many students (median 63%, IQR 63–87%) had encountered an ADR during their clinical training [41, 44, 45, 48, 58], only a few (median 10%, IQR 13%) had previously been involved in reporting an ADR [39–42, 45, 58, 63]. Most students did not know where to report an ADR (median 57%, IQR 47–91%) [37, 39, 40, 46–48, 56], which method they should be used to report an ADR (median 72% IQR 62–86%) [38, 41, 42, 45, 46], or how to get access to the ADR report form (median 84%, IQR 61–92%) [41–43, 48, 54].

Sixteen studies analyzed students’ opinions of their perceived level of training in pharmacovigilance and ADR reporting (Supplement Fig. 1). One study of pharmacy students concluded that students felt sufficiently trained [37]. Conversely, six studies of pharmacy and medical students showed that students felt inadequately qualified to report ADRs or to perform pharmacovigilance [41–44, 48, 51]. Additionally, three (27%) studies reported that fourth- and fifth-year medical and pharmacy students also felt to have inadequate knowledge to report ADRs [42, 49, 51]. Healthcare students in almost all (15 studies) studies felt that ADR reporting and pharmacovigilance should be included in pharmacy and medical curricula [38, 40–43, 45, 47, 48, 53, 57, 59, 64–66]. Two studies reported that dentistry [46] and nursing [56] students felt neither positive nor negative about including ADR reporting in their curriculum.

Seven studies individually analyzed student reasons for reporting or *not* reporting ADRs to the competent authority [6, 37, 39, 40, 43, 47, 67] (Supplement Table 3). A lack of encouragement (*n* = 3), lack of information provided by patients (*n* = 2), and a lack of knowledge on how to report (*n* = 2) were the reasons most often given for *not* reporting ADRs. Educating others (*n* = 3), improving patient safety (*n* = 3), and contributing to the safe use of medicines (*n* = 3) were the reasons most often given for reporting ADRs.

### What factors influence pharmacovigilance competencies?

Two comparative studies investigated differences in attitude and knowledge to pharmacovigilance and ADR reporting between medical and pharmacy students [47, 57]. Sivadasan et al. [45, 57] showed that more medical students than pharmacy students considered ADR reporting to be essential (80.5 vs 75.8%) and considered it their professional responsibility (69 vs 51.6%) [45]. Conversely, Umair Khan et al. showed that significantly more pharmacy students than medical students considered ADR reporting as important as managing patients (79.1 vs 43.5%) [47]. Both studies concluded that final-year pharmacy students had superior pharmacovigilance knowledge compared with medical students: 5.61 ± 1.78 vs 3.23 ± 1.60, 0–10 min/max and 8.4 ± 0.2 vs 3.17 ± 0.06; 0–15 min/max, respectively [47, 57].

Additional comparisons between gender, race/ancestry, pharmacology curricula, previous pharmacovigilance or ADR-reporting training, previous ADR-reporting experience, and level of professional year were analyzed to identify factors associated with a higher level of pharmacovigilance competence. Race/ancestry did not influence pharmacovigilance knowledge, although male students knew more about post-marketing surveillance and female students knew more about causality assessments [38]. Overall, PharmD (Master of Pharmacy) students had more positive attitudes and higher knowledge scores than BPharm (Bachelor of Pharmacy) students [37, 43], probably because the former had trained for longer. A positive correlation was found between student knowledge and their skills in ADR reporting (*r* 0.485, *p* < 0.001) [50]. Previous training in ADR reporting or reporting experience was associated with significantly higher student knowledge scores [40, 47, 53]. In line with these observations, academically older students had more knowledge, were more aware of ADRs during their internships, and had reported more ADRs.

### Which pharmacovigilance interventions are effective?

There is no uniform pharmacovigilance educational intervention (Table 2). Interventions have ranged from short 15-min power point lectures and multiple training workshops to more innovative clinical experiences in ADR reporting or assessment. No replicated intervention studies have been published to our knowledge.

**Table 2:** Articles (*n* = 14) evaluating pharmacovigilance intervention studies in undergraduate healthcare students

Author	Country	Student type	Total students	Intervention type	Quantitative description	Measurement instrument	Follow-up	Kirkpatrick level^a^	Conclusion
Arici et al. [68]	Turkey	Medical (fifth year)	77	Theoretical information and ADR-reporting practice	One session of 2 h	Questionnaire	Direct and after 12 months	2b	Significant increase in short-term knowledge score without an impact in the long-term.
Amarnath et al. [69]	India	Pharmacy and nursing (second to fourth year)	213	Interactive power point lecture	One lecture of 45 min	Questionnaire	Direct	2b	Nursing students had a better overall knowledge of pharmacovigilance than pharmacy students. However, they lacked awareness regarding documentation.
Armando et al. [70]	Argentina	Pharmacy (second year)	50	Identification of ADRs	?	Number of identified ADRs	–	4a	Students were equally capable of recognizing ADRs in a community setting as pharmacists.
Chandy et al. [71]	India	Medical (second to third year)	88	Medication safety module	One lecture of 2 h	Questionnaire	After 1 month	2b	Significant increase in the pre-existing poor medication safety knowledge score (9.52 ➔ 12.24 out of 20).
Christensen et al. [72]	Denmark	Pharmacy (fourth year)	13	Detection of ADR by questioning medication users	?	Number of reported ADRs	–	4a	Community pharmacy interns were capable of detecting and reporting ADRs (33 out of 128 patients reported 45 ADRs).
Durrieu et al. [73]	France	Medical (third year)	92	General pharmacology courses	Part of a total session of 74 h	Visual analogue scale	Direct	2a	Pharmacological training allows students to be aware of potentially serious ADRs associated with drugs, in particular with drugs considered relatively safe, such as NSAIDs and aspirin.
Durrieu et al. [74]	France	Medical (fifth year)	67	General pharmacology course	2 year of clinical training	Visual analogue scale	36 months	2a	Risk perception of ADRs was modified after clinical training: still aware of potentially serious ADRs related to anticoagulants, aspirin, or NSAIDs, less cautious about antidepressants
Mohan et al. [65]	India	Medical (second year)	56	Training workshop	Three sessions of 30 min	Questionnaire	Direct	2a	Positive evaluation of the workshop and the sessions created pharmacovigilance awareness.
Naritoku et al. [75]	USA	Medical (fourth year)	61	Advanced therapeutics course	Part of a total session of 90 h	Questionnaire	Direct	1	The course structure appeared useful for educating students about therapeutics that lacked a sufficient clinical pharmacology faculty.
Reddy et al. [66]	India	Pharmacy (fourth to sixth year)	225	Interactive educational intervention program	One session (time unknown)	Questionnaire	Direct	2b	Significant increase in student knowledge score (e.g., over 15% more students knew to what the study of pharmacovigilance related).
Rosebraugh et al. [60]	USA	Medical (fourth year)	78	Lecture on completing a MedWatch form	One session of 15 min	Quality score of ADR report	Direct	2b	Significant improvement in the quality of completing a fictional ADR-report.
Schutte et al. [67]	Netherlands	Medical (first to fifth year)	43	Assessment of ADR reports	On average 3 times (total time 12 h)	Quality of ADR-assessment and questionnaire	Direct	4a	Students were capable of high-quality assessments of ADR reports without costing staff from a pharmacovigilance center extra time.
Sullivan et al. [76]	USA	Pharmacy (second to third year)	26	Student ADR-reporting program	?	Number of reported ADRs	–	4a	Significant increase in the number (42 → 310) of ADRs documented.
Tripathi et al. [77]	India	Medical (second year)	180	Working group on ADR reporting and monitoring	One working group (time unknown)	Quality score of ADR report	After 1 and 6 months	2b	Significant increase in ADR-reporting skills after 1 and 6 months.

*ADRs*, adverse drug reactions

^a^Kirkpatrick’s four levels of training evaluations are as follows: Level 1—participation: covers learners’ views on the learning experience, its organization, presentation, content, teaching methods, and aspects of the instructional organization, materials, and quality of instruction; Level 2a—modification of attitudes and perceptions: outcomes relate to changes in the reciprocal attitudes or perceptions between participant groups toward the intervention or simulation; Level 2b—modification of knowledge or skills: for knowledge, this relates to the acquisition of concepts, procedures, and principles; for skills, this relates to the acquisition of thinking problem solving, psychomotor, and social skills; Level 3—behavioral change: documents the transfer of learning to the workplace or willingness of learners to apply new knowledge and skills; Level 4a—change in organizational practice: wider changes in the organization or delivery of care, attributable to an educational program; Level 4b—benefits to patient or clients: this relates to any improvement in the health or well-being of patients clients as a direct result of an educational program

Four articles evaluated student satisfaction regarding pharmacovigilance education [65, 67, 75, 77]. Students found clinical experience more educational than lectures and/or solving fictional casuistry [67]. Students also stressed that pharmacovigilance training should be repeated during the internships [77]. Six articles examined students’ intentions and attitudes toward ADR reporting after a pharmacovigilance intervention [65–67, 73, 74, 77]. However, since none of the studies included a baseline assessment and substantial differences were not observed between cross-sectional and intervention studies, it was not possible to draw conclusions.

Two studies by Durrieu et al. focused on students’ perception of the risk of ADRs [73, 74]. They concluded that after a pharmacology course, students were more aware of potentially serious ADRs. A follow-up study showed that perception of the risks of ADRs was more clinically realistic after clinical training, i.e., students were more aware of potentially serious ADRs associated with anticoagulants and non-steroidal anti-inflammatory drugs (NSAIDs) and less conservative about hypercholesterolemia drugs.

Five studies showed a significant increase in pharmacovigilance and ADR-reporting knowledge scores directly after the intervention was completed [66–69, 71]. Since most studies asked different pharmacovigilance questions or used grouped outcome scores [68, 69, 71], it was not possible to state that one intervention was superior to another. Studies with a longer follow-up time (1–12 months) reported contrasting outcomes. Two studies showed a significant increase in pharmacovigilance knowledge and ADR-reporting skills after 1 and 6 months [71, 77]. However, Arici et al. reported a significant increase in pharmacovigilance knowledge directly after an intervention, but this had faded by 12 months [68].

Three studies analyzed pharmacovigilance or ADR-reporting skills [60, 67, 77]. Schutte et al. showed that medical students were significantly more aware of the importance of ADR reporting after assessing a real ADR report themselves [67]. Tripathi et al. and Rosebraugh et al. analyzed the impact of an intervention on the quality of completing a fictional ADR report in undergraduate medical students [60, 77]. Both showed that a 15-min lecture significantly increased the quality of an ADR report.

Four articles analyzed pharmacovigilance competences in a real-life clinical setting [67, 70, 72, 76], three of which involved pharmacy students [70, 72, 76]. Findings suggested that pharmacy students could play an important part in regular pharmacovigilance healthcare. Christensen et al. and Sullivan and Spooner found a significant increase in the number of ADRs reported in a hospital setting [72, 76], and Armando et al. found that second-year pharmacy students were equally capable of recognizing ADRs in a community pharmacy setting as pharmacists [70]. Schutte et al. showed that medical students were also capable of assessing real ADR reports [67].

## Discussion

We found that while healthcare students have favorable intentions and positive attitudes toward ADR reporting, most lack the basic skills and knowledge to do so. Overall, academically older students and students with prior pharmacovigilance experience were more competent in recognizing and reporting ADRs. Pharmacy students had slightly more knowledge of pharmacovigilance and ADR reporting than other healthcare students. Students agreed that pharmacists are the most important healthcare professional with regard to pharmacovigilance, although all students felt responsible for pharmacovigilance. Students perceived their knowledge to be moderate at best, felt they did not receive sufficient training, and stated that pharmacovigilance and ADR reporting should be included in their curriculum. It is not surprising that while relatively many students had seen an ADR (63%), few had reported one (10%). This is consistent with previous studies [78] and the current low rate of ADR reporting (medial reporting rate of 6%) [29] among qualified health professionals.

Despite this lack of competence in pharmacovigilance and ADR reporting, we identified 14 studies that reported beneficial effects of an intervention. Students valued real and legitimate pharmacovigilance tasks, such as diagnosing, reporting, or assessing ADR reports, more than outdated educational interventions or fictional casuistry. This type of clinical training also leads to a more clinically realistic perception of the risk of ADRs. Although educational pharmacovigilance interventions ultimately aim at a clinically relevant and long-term increase in medication safety, no study has looked at this highest hierarchical level. Most outdated interventions only provide a short-term increase in knowledge, few show clinically relevant results, and none has shown durable clinical outcomes. Repeated clinical training which boosts intrinsic motivation and improves learning outcomes [79, 80] should be applied to pharmacovigilance training. Additionally, the interventions that focused on real and legitimate clinical tasks, such as diagnosing and reporting ADRs and assessing ADR reports, also had a positive effect on the healthcare system. Multiple studies have shown the clinical value of student participation in pharmacovigilance tasks.

Although our findings are worrying, the outcome should be interpreted with some caution given the heterogeneity and methodological weaknesses of the included studies. All intervention studies were single institution, had variable intervention designs, used different assessment methods of no clear relevance, and were ultimately of moderate study quality (mean MERSQI score 11.1). Since this is the first systematically performed review to investigate the current pharmacovigilance competencies of all types of healthcare students, we cannot compare our findings with those of other studies. A similar review, focusing on only a few competencies in medical students, reported similar outcomes [5].

This review had a number of limitations. Articles may have been missed, although we attempted to reduce the likelihood of this by searching six databases and using a snowball strategy. Overall, the studies were only of moderate quality, with low response rates, and small intervention groups, many of which had not been retested. Despite these weaknesses and the possibility that student capabilities were overestimated, because of publication bias, most competencies are still far from satisfactory. Moreover, the heterogeneity of assessment instruments used, outcome measures, and interventions, in combination with the combined competency scores in some studies, made a full comparison or meta-analysis impossible. However, this heterogeneity could mask some interesting features, since only few frequently reported variables were studied in detail. Lastly, the difference in location of cross-sectional studies (66% in Asia) and intervention studies (24% in Asia) may have skewed the analyses.

## Conclusion

This review highlights the urgent need to improve and modernize current pharmacovigilance education for undergraduate healthcare students. However, the best way to provide this education still needs to be established, but the content of pharmacovigilance education should at least be as real as possible. We suggest it is given real life context, i.e., with clinical relevance as early responsibility for the student (under supervision). It should be integrated into different healthcare curricula (medicine, pharmacy, dentistry, and nursing) and repeated throughout academic training, starting as early as possible, in the Bachelor phase. By offering real clinical pharmacovigilance training, students can not only increase their knowledge, awareness, and skills, but can also assist current healthcare professionals meet their clinical pharmacovigilance obligations. Future research should therefore focus on valid and reliable methods for assessing pharmacovigilance competencies in clinical practice. To successfully develop and initiate pharmacovigilance educational programs, further work is needed to evaluate educational interventions on Kirkpatrick’s highest hierarchical levels, preferably in an inter-professional setting, with a multicenter design and a long follow-up. Internships or student-run clinics may be useful since they offer students early pharmacovigilance experiences with real responsibilities for patient care, with the advantage of assisting current healthcare professionals, limiting the level of underreporting, and ultimately preventing ADRs and increasing patient safety.

## Electronic supplementary material

ESM 1 (DOCX 33 kb)
