# Supplementary material for: Urgent need to modernize pharmacovigilance education in healthcare curricula: review of the literature
Source: Eur J Clin Pharmacol. 2018 Jun 20;74(10):1235–48. doi: 10.1007/s00228-018-2500-y (PMC6132536; doi:10.1007/s00228-018-2500-y)
Supplement: Supplementary file 1 — (DOCX 33 kb) [file 228_2018_2500_MOESM1_ESM.docx]

**Supplementary document**

**Supplementary texts – Results**

- Study characteristics
- Study quality

**Supplement figure 1**: Student opinions on: received training, current knowledge, and need for further pharmacovigilance and ADR-reporting training.

**Supplement table 1**: Inclusion and exclusion criteria.

**Supplement table 2**: MERSQI subdomain and total mean scores.

**Supplement table 3**: Students’ reasons for (not) reporting ADRs to the competent authority.

**Search quiries:** Search strategy performed in MEDLINE (PubMed), EMBASE and ERIC databases on 10 January 2017.

# Supplementary texts - Results

# Study characteristics

The number of articles evaluating pharmacovigilance competencies has increased substantially in the last decade, with 8 studies being published before 2010 and 31 in the last 7 years; however, the proportion of pharmacovigilance intervention studies has decreased compared with the number of cross-sectional studies (<2010: 50% intervention studies, >2010: 29% intervention studies). Of the included articles, most came from Asia (n=25), followed by America (n=7), Europe (n=5), and Africa (n=2). However, the proportion of intervention studies compared to the number of cross-sectional studies was skewed: Europe (n=4 of 5), America (n=4 of 7), Asia (n=6 of 25), and Africa (n=0 of 2). The studies involved students predominately in the 4^th^ (31%) and 5^th^ academic year (28%) of their medical (n=22; 46%) or pharmacy (n=22; 46%) curriculum. The competencies of nursing and dentistry students on pharmacovigilance were investigated in two studies.

# Study quality

The total MERSQI scores of the articles ranged from 6 to 14.5, with a mean (SD) of 11.09 (2.41). Eight articles were of high methodological quality (MERSQI score ≥13.5) and 7 were of low methodological quality (MESQI-score ≤8.5). The total mean MERSQI score of articles measuring “opinions and attitudes” as primary outcome (Kirkpatrick level 1 and 2a) was significantly lower than that of articles measuring “knowledge and skills” (Kirkpatrick level 2b) (p<0.001) or “healthcare outcomes” (Kirkpatrick level 4a) (p<0.016) in a one-way ANOVA (Supplement Table 2). No significant difference was found between the total mean MERSQI score in articles measuring “knowledge and skills” (Kirkpatrick level 2b) and “healthcare outcomes” (Kirkpatrick level 4a). Of the 39 articles, 10 (25.6%) were classified as having a multi-institution design and were all cross-sectional studies. One third (n=14, 35%) of all articles did not report their response rate and lacked overall validity. However, almost all articles (n=31, 77.5%) used objective data and the majority (72.5%) used analytical methods other than descriptive analysis (Supplement Table 2).

# Supplement figure 1

**Supplement figure 1:** Average student opinions regarding: received training, current knowledge, and need for further pharmacovigilance and ADR-reporting training in undergraduate healthcare students.

# Supplement table 1

|  | Inclusion criteria | Exclusion criteria |
| --- | --- | --- |
| Study evaluation | Evaluation of general pharmacovigilance competencies | Evaluation of a (specialty) specific ADR or only studying medical or dietary supplements, herbal products or alternative medicines |
| Population of interest | Undergraduate healthcare students: medicine, pharmacy, dental and nursing | Non-healthcare students (e.g. healthcare professionals or patients) |
| Study types and designs | Original quantitative and qualitative published studies | Non-original studies: reviews, editorials, letters to the editor and conference abstracts |
| Outcome measure | At least on pharmacovigilance competences based on the Kirkpatrick hierarchy model |  |
| Publication dates | Until Jan 1 2017 |  |
| Publication language | Dutch or English |  |

**Supplement Table 1**: Inclusion and exclusion criteria for studies on undergraduate pharmacovigilance education.

# Supplement table 2

|  | |  | Attitudes, opinions and general facts | Knowledge and skills | Healthcare outcome | Total |
| --- | --- | --- | --- | --- | --- | --- |
| Articles, n (%) | | | 8 (20.5%) | 27 (69.2%) | 4 (10.3%) | 39 (100%) |
| MERSQI subdomain design | | | | | | |
| Study design | | |  |  |  |  |
|  | **Cross-sectional** | | 6 (15.4%) | 21 (53.8%) | 3 (7.7%) | 30 (76.9%) |
|  | **Single-group, pre- and post- test** | | 2 (5.1%) | 4 (10.2%) | 1 (2.6%) | 7 (17.9%) |
|  | **Non-randomized** | | 0 | 1 (2.6%) | 0 | 1 (2.6%) |
|  | **Randomized controlled trial** | | 0 | 1 (2.6%) | 0 | 1 (2.6%) |
| MERSQI subdomain sampling | | | | | | |
| Institutions | | |  |  |  |  |
|  | **1 institution** | | 6 (15.4%) | 20 (51.3%) | 4 (10.2%) | 30 (76.9%) |
|  | **2 institutions** | | 0 | 0 | 0 | 0 |
|  | **>2 institutions** | | 2 (5.1%) | 7 (17.9%) | 0 | 9 (23.1%) |
| Response rate | | |  |  |  |  |
|  | **Not applicable** | |  |  |  |  |
|  | **Not reported** | | 1 (2.6%) | 10 (25.6%) | 3 (7.7%) | 14 (35.9%) |
|  | **<50%** | | 1 (2.6%) | 2 (5.1%) | 0 | 3 (7.9%) |
|  | **50-74%** | | 1 (2.6%) | 8 (20.5%) | 1 (2.6%) | 10 (25.6%) |
|  | **≥75%** | | 5 (12.8%) | 7 (17.9%) | 0 | 12 (30.7%) |
| MERSQI subdomain data | | | | | | |
| Type of data | | |  |  |  |  |
|  | **Assessment by study participant** | | 8 (20.5%) | 1 (2.6%) | 0 | 9 (23.1%) |
|  | **Objective measurement** | | 0 | 26 (66.7%) | 4 (10.2%) | 30 (76.9%) |
| MERSQI subdomain validity of evaluation instrument | | | | | | |
| Internal structure | | |  |  |  |  |
|  | **Reported** | | 1 (2.6%) | 12 (30.8%) | 0 | 13 (33.3%) |
|  | **Not reported** | | 7 (17.9%) | 15 (38.5%) | 4 (10.2%) | 26 (66.7%) |
| Content | | |  |  |  |  |
|  | **Reported** | | 3 (7.7%) | 23 (59%) | 1 (2.6%) | 27 (69.2%) |
|  | **Not reported** | | 5 (12.8%) | 4 (10.2%) | 3 (7.7%) | 12 (30.8%) |
| Relationships to other variables | | |  |  |  |  |
|  | **Reported** | | 3 (7.7%) | 23 (59%) | 2 (5.1%) | 28 (71.8%) |
|  | **Not reported** | | 5 (12.8%) | 4 (10.2%) | 2 (5.1%) | 11 (28.2%) |
| MERSQI subdomain data analysis | | | | | | |
| Complexity of analysis | | |  |  |  |  |
|  | **Descriptive only** | | 5 (12.8%) | 3 (7.7%) | 3 (7.7%) | 11 (28.2%) |
|  | **Beyond descriptive** | | 3 (7.7%) | 24 (61.5%) | 1 (2.6%) | 28 (71.8%) |
| Appropriateness of analysis | | |  |  |  |  |
|  | **Appropriate** | | 5 (12.8%) | 22 (56.4%) | 3 (7.7%) | 30 (76.9%) |
|  | **Not appropriate** | | 3 (7.7%) | 5 (12.8%) | 1 (2.6%) | 9 (23.1%) |
| MERSQI total score, mean ± SD | | | | |  |  |
|  |  | | **7.81 ± 1.69** | **12.02 ± 1.67** | **11.30 ± 2.50** | **11.10 ± 2.41** |

**Supplement Table 2**: MERSQI subdomain and total mean scores or characteristics of three outcomes (i.e. attitudes and opinions, skills and knowledge, and patient and health care)

# Supplement table 3

| Reason for not reporting | Number of articles  (out of 3) |
| --- | --- |
| Lack of encouragement / ADR-reporting culture | 3 |
| Lack of information provided by patients | 2 |
| I do not know where and how to report | 2 |
| Patient overload | 1 |
| Reporting is not mandatory | 1 |

| Reason for reporting | Number of articles  (out of 4) |
| --- | --- |
| Contributes to the safe use of medicines | 3 |
| Improves patient safety | 3 |
| Educates others about drug risks | 3 |
| To measure the incidence of ADR’s | 1 |
| Personally benefitting | 1 |
| To identify factors that might predispose to an ADR | 1 |

**Supplement Table 3a/3b**: Students’ reasons for (not) reporting ADRs to the competent authority.

# Search queries

**PUBMED** (n=980)

((((Pharmacovigilance*[tiab]) OR "Pharmacovigilance"[Mesh]) OR "Adverse Drug Reaction Reporting Systems"[Mesh])) AND ((("Students"[Mesh]OR student*[tiab]OR undergraduate*[tiab]) OR ((Educat*[tiab] OR teaching*[tiab] OR training*[tiab] OR learn*[tiab] OR curricul*[tiab]))) OR (("Education"[Mesh]) OR "education" [Subheading] OR "Educational Status"[Mesh]))

**EMBASE** (n=1965)

**pharmacovigilan***:ab,ti OR **'pharmaco vigilan*'**:ab,ti **OR ‘adverse drug reaction reporting systems’/exp AND 'education'**/exp OR **'educational status'**/exp OR **'student'**/exp OR **educat***:ab,ti OR **teaching***:ab,ti OR **training***:ab,ti OR **learn***:ab,ti OR **curricul***:ab,ti OR **competenc***:ab,ti OR **student***:ab,ti OR **undergraduate***:ab,ti

**Cochrane database** (n=111: 62 Cochrane reviews, 45 trials, 1 method, 2 other reviews, 1 economic evaluation)

pharmacovigilan*:ab,ti,kw OR "pharmaco-vigilan*":ab,ti,kw OR adverse drug reaction reporting system*:ab,ti,kw AND Educat*:ti,ab,kw OR teaching*:ti,ab,kw OR training*:ti,ab,kw OR learn*:ti,ab,kw OR curricul*:ti,ab,kw OR competenc*:ti,ab,kw OR student*:ti,ab,kw OR undergraduate*:ti,ab,kw

**CINAHL** (n=42)

MH "Pharmacovigilance" OR TI (Pharmacovigilan* OR "Pharmaco-vigilan*" OR Adverse drug reactions reporting systems) OR AB (Pharmacovigilan* OR "Pharmaco-vigilan*" OR Adverse drug reactions reporting systems) AND (MH "Education+") OR (MH "Educational Status") OR (MH "Students+") OR TI (Educat* OR teaching* OR training* OR learn* OR curricul* OR competenc*) OR AB (Educat* OR teaching* OR training* OR learn* OR curricul* OR competenc*) OR TI (student* OR undergraduate*) OR AB (student* OR undergraduate*)

**ERIC** (n=1)

TI (Pharmacovigilance OR "Pharmaco-vigilance" OR Adverse drug reactions reporting systems) OR AB (Pharmacovigilance OR "Pharmaco-vigilance" OR Adverse drug reactions reporting systems) AND (DE "Academic Settings" OR DE "Curriculum" OR DE "Undergraduate Education" OR DE "Schools" OR DE "Education" OR DE "Nursing Education" OR DE "Teaching" OR DE "Training") OR (DE "Students" OR DE "Medical Students" OR DE "Dental Students") OR TI (Educat* OR teaching* OR training* OR learn* OR curricul* OR competenc*) OR AB (Educat* OR teaching* OR training* OR learn* OR curricul* OR competenc*) OR TI (student* OR undergraduate*) OR AB (student* OR undergraduate*)

**PsycInfo** (n=19)

TI (Pharmacovigilance OR "Pharmaco-vigilance" OR Adverse drug reactions reporting systems) OR AB (Pharmacovigilance OR "Pharmaco-vigilance" OR Adverse drug reactions reporting systems) AND (DE "Academic Settings" OR DE "Curriculum" OR DE "Undergraduate Education" OR DE "Schools" OR DE "Education" OR DE "Nursing Education" OR DE "Teaching" OR DE "Training") OR (DE "Students" OR DE "Medical Students" OR DE "Dental Students") OR TI (Educat* OR teaching* OR training* OR learn* OR curricul* OR competenc*) OR AB (Educat* OR teaching* OR training* OR learn* OR curricul* OR competenc*) OR TI (student* OR undergraduate*) OR AB (student* OR undergraduate*
